# Evolution and molecular interactions of major histocompatibility complex (MHC)-G, -E and -F genes

**DOI:** 10.1007/s00018-022-04491-z

**Published:** 2022-08-04

**Authors:** Antonio Arnaiz-Villena, Fabio Suarez-Trujillo, Ignacio Juarez, Carmen Rodríguez-Sainz, José Palacio-Gruber, Christian Vaquero-Yuste, Marta Molina-Alejandre, Eduardo Fernández-Cruz, José Manuel Martin-Villa

**Affiliations:** 1 Departamento de Inmunología, Facultad de Medicina, Universidad Complutense de Madrid, Pabellón 5, planta 4. Avda. Complutense s/n, 28040 Madrid, Spain; 2 Instituto de Investigaciones Sanitarias Gregorio Marañón, Hospital Gregorio Marañón, Madrid, Spain

**Keywords:** MHC, Evolution, HLA-G, HLA-E, HLA-F, Complotypes, Haplotypes, Disease, HLA, Apes, Monkeys

## Abstract

Classical *HLA* (Human Leukocyte Antigen) is the Major Histocompatibility Complex (MHC) in man. HLA genes and disease association has been studied at least since 1967 and no firm pathogenic mechanisms have been established yet. *HLA-G* immune modulation gene (and also *-E* and *-F*) are starting the same arduous way: statistics and allele association are the trending subjects with the same few results obtained by *HLA* classical genes, i.e., no pathogenesis may be discovered after many years of a great amount of researchers’ effort. Thus, we believe that it is necessary to follow different research methodologies: (1) to approach this problem, based on how evolution has worked maintaining together a cluster of immune-related genes (the MHC) in a relatively short chromosome area since amniotes to human at least, i.e., immune regulatory genes (MHC-G, -E and -F), adaptive immune classical class I and II genes, non-adaptive immune genes like (C2, C4 and Bf) (2); in addition to using new in vitro models which explain pathogenetics of *HLA* and disease associations. In fact, this evolution may be quite reliably studied during about 40 million years by analyzing the evolution of *MHC-G, -E, -F*, and their receptors (KIR—killer-cell immunoglobulin-like receptor, NKG2—natural killer group 2-, or TCR-T-cell receptor—among others) in the primate evolutionary lineage, where orthology of these molecules is apparently established, although cladistic studies show that *MHC-G* and *MHC-B* genes are the ancestral class I genes, and that New World apes *MHC-G* is paralogous and not orthologous to all other apes and man *MHC-G* genes. In the present review, we outline past and possible future research topics: co-evolution of adaptive *MHC* classical (class I and II), non-adaptive (i.e., complement) and modulation (i.e., non-classical class I) immune genes may imply that the study of full or part of MHC haplotypes involving several loci/alleles instead of single alleles is important for uncovering HLA and disease pathogenesis. It would mainly apply to starting research on HLA-G extended haplotypes and disease association and not only using single HLA-G genetic markers.

## Physiopathology

### The non-classical class I HLA genes: HLA-G, -E, and -F

**Fig. 1:**
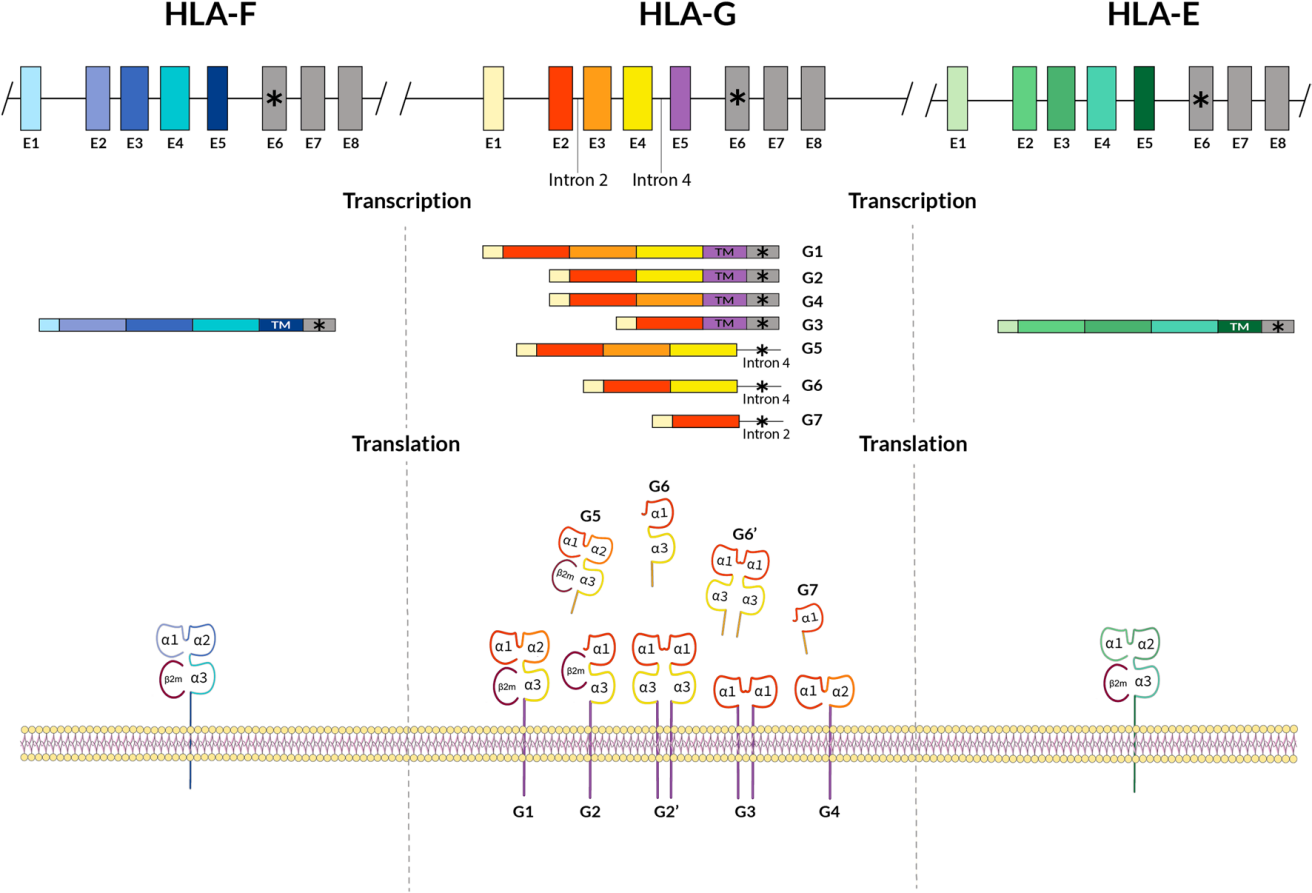
HLA gene complex is located in the short arm of human chromosome 6 (6p21.3). HLA-G, -E and -F mRNA transcription and translation scheme and HLA-G membrane and soluble isoforms are shown (see text). Exons (E) of each gene are shown in upper panels of the figure. A (*) symbol indicates a stop codon: it may be localized in E6 in HLA-E, -F and -G genes. HLA-G also presents stop codons in intron 2 or intron 4 depending on alternative splicing process which gives rise to different isoforms. Stop codon may be maintained in mature mRNA due to a reading-through mechanism in humans and primates which is described also in other HLA genes (i.e., HLA-DRB6). The presence of a selenocysteine insertion sequence (SECIS) at the 3 untranslated region leads to a selenocysteine incorporation at UGA (stop) codons [15–18]; this may be the cause for stop codon maintenance in HLA-G, -E and -F translation. Beta-2 microglobulin (β2m) is represented bound to protein molecules in purple color. See also references [19, 20]

The human Major Histocompatibility Complex is a genomic region which comprises at least 224 genes at chromosome 6p21.3, coding for the so-called *HLA* complex (counterpart to *MHC* in other vertebrates) that has a key role on the immune system. Classical class I genes (*HLA-A, HLA-B,* and *HLA-C*) encode for molecules that present antigen peptides to clonotypic T-cell receptor on the surface of CD8 + cells, whereas the non-classical class I proteins (HLA-G, HLA-E, and HLA-F) (Fig. 1) have been primarily associated with the modulation of the immune system cells [1–3]. HLA-G was first considered to be an immune modulatory molecule, predominantly expressed at the maternal–fetal interface and its function was first assigned to *maternal–fetal tolerance* [2, 4–6]. Initial studies were carried out by Dan Geraghty et al. [7] and they named *HLA-6.0* the new gene they described. HLA-6.0 protein was structurally similar to HLA-A, -B, and -C class I molecules but with a premature in-frame stop codon that hindered translation of an important part of the cytoplasmatic region in HLA-6.0 mature molecule. The promoter region of *HLA-6.0* gene was similar to that of *MHC-Qa* mouse gene, and both genes were equivalent with regard to substitutions, deletions and other variations in allelic DNA sequences [7]. Warner et al. group [8] proposed that *MHC-Qa* was a functional *HLA-G* homologue in mouse, with a similar gene and protein structure; MHC-Qa also presents soluble forms like HLA-G5, G6 and G7 isoforms in humans (Fig. 1). Recently, it is found that *Qa-1b*(MHC-Qa non-classical class I gene in mouse) seems to be homologous to *HLA-E* (see HLA-E “Evolution” section). The complete HLA-G molecule has an extracellular structure very similar to that of the classical HLA molecules, though its major function is not antigen presentation. It was found that HLA-G inhibits the cytotoxic activity of T CD8 + and NK cells through direct interaction with leukocyte receptors, such as LILRB1 (LIR1/ILT2), LILRB2 (ILT4), and KIR2DL4 (CD158d) [3, 9–14].

**Fig. 2:**
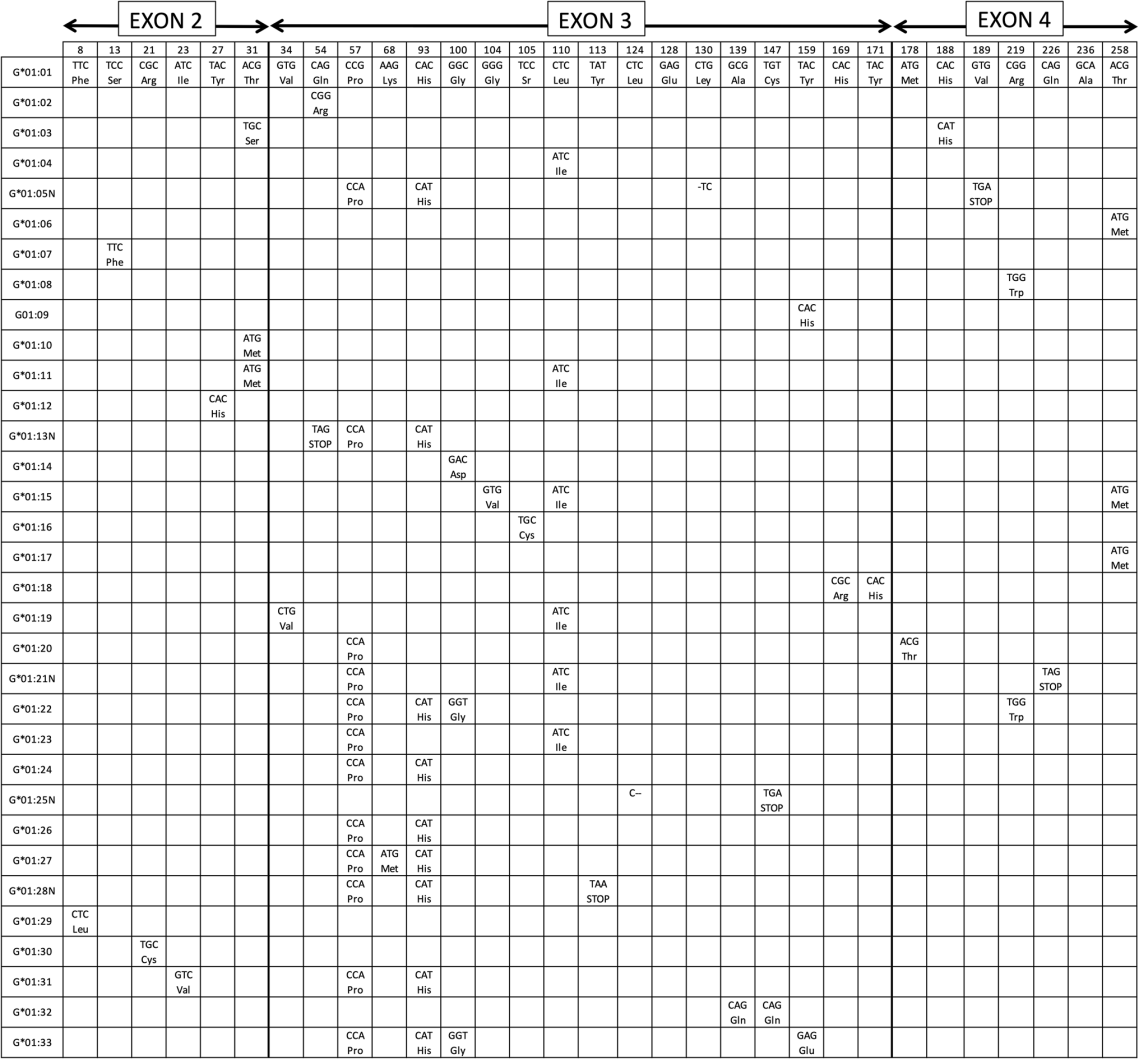
HLA-G protein alleles. Codon and aminoacidic changes among different alleles in exon 2, exon 3 and exon 4 are shown. The letter “N” at the end of some alleles shown in the table denotes null allele. These null alleles bear a stop codon due to single-base deletions or point mutation which give rise to an incomplete HLA-G protein translation. HLA-G*01:05N has a single cytosine deletion at codon 130 (CTG → TGC) which produces a reading frameshift change, causing a premature stop signal at codon 189 (GTG → TGA) [38, 39] and consequently a shorter protein with α1 functional domain at least [38, 40]. HLA-G*01:21N has a premature stop codon due to a punctual mutation in codon 226 (CAG → TAG) of coding sequence which leads to a non-complete translated protein [40]. The number of HLA-G protein alleles is rapidly growing; see IMGT-HLA database to be up to date on new alleles (https://www.ebi.ac.uk/ipd/imgt/hla; accessed September 2021) [41]

*HLA-G* gene and molecule expression patterns differ in many aspects compared to classical HLA class I molecules, like: (a) a restricted tissue expression in normal conditions [21]; it is being expressed on the maternal–fetal interface in the extravillous cytotrophoblast cells [6], cornea, proximal nail matrix, thymus, hematopoietic stem cells and pancreas mainly [22–27]. HLA classical class I molecules (HLA-A, -B, and -C) are widely expressed in all body tissues. Non-classical class I HLA molecules (HLA-E, -F, and -G) are more restricted regarding tissue localization, antigen presentation, and function [3]. Diversity of presented peptides compared with that of classical class I MHC molecules is much reduced probably because of their limited levels of polymorphism [28]. These non-classical class I molecules may also regulate immunity through TCR-independent interactions (see below); (b) they show several membrane and soluble isoforms due to alternative splicing of the complete *HLA-G* mRNA [2, 3]; (c) a short cytoplasmic tail is present due to the presence of a premature stop codon at exon 6 [2, 3]; (d) a relatively low HLA-G protein polymorphism is recorded although it is rapidly increasing (Fig. 2) [2, 3, 29]; (e) they present a unique 5’URR (5’ upstream regulatory region) different from other HLA classical class I genes [30, 31]; and (f) the 5’ promoter region [2, 32–36] and the 3’UTR (3’ untranslated region) show several polymorphisms that are specifically linked to diseases susceptibility [37].

Also, it has been shown that HLA-G presents endogenous peptides at the surface of the placenta trophoblast [42], absent in other HLA classical class I molecules’ expression [43], with the exception of HLA-C [44]. Thus, HLA-G interacts at this maternal–fetal interface with activating and inhibitory receptors: killer-cell immunoglobulin-like receptor (KIR), leukocyte immunoglobulin-like receptor (LIR), and CD94-NKG2 receptor complex families to establish maternal tolerance and normal fetal growth [43]. This non-classical class I HLA molecule recognizes TCR of regulatory [45] and cytolytic [46] CD8 T cells [47].

On the other hand, *HLA-E* polymorphism is represented only by two functional molecules that present a set of similar peptides derived from class I leader sequences. However, HLA-E is a ligand for the innate and adaptive immune system effectors; immunological response to peptide-HLA-E complexes is determined by the sequence of the bound peptide, which interacts with CD94/NKG2 or T-cell receptor [48, 49].

While HLA-E and HLA-G have been well-characterized functionally and structurally, the role that HLA-F plays in regulating the immune system has long time been unknown. However, HLA-F has been shown to protect fetus development [50] and has a role at peripheral nervous system: HLA-F recognition by the inhibitory KIR3DL2 receptor prevents motor neuron death in amyotrophic lateral sclerosis physiopathology [51]. Also, HLA-F interacts with the activating KIR3DS1 on NK cells and induces an anti-viral response against HIV-1 (human immunodeficiency virus-1) [52]. HLA-F immunity regulation by KIR3DS1 interaction has increased the clinical importance of HLA-F since also other diseases exist where KIR3DS1 has a pathogenetic role [53]. Thus, HLA-F and disease relationship is important but the molecule structural and biochemical properties and the precise relationship with its function is mostly unknown. “In-silico” studies predicted that HLA-F has the typical MHC fold but with only a partially open-ended groove [47, 54].

### Role of MHC-G, -E and -F as immune-regulation proteins: pathology

Expression of *HLA-G* has been studied in autoimmune and inflammatory diseases, tumors, chronic viral infections and in engrafted tissues [5, 55–58]. This *HLA-G* expression has been associated with better prognosis in chronic inflammation, autoimmune diseases, and allotransplants, because inhibition of immune response occurs; however, this inhibition may be harmful in chronic viral infections and tumors, where an efficient immune response may be hindered [59, 60]. The role and pathology of MHC-G, -E, and -F in maternal/fetal relationship has been widely reviewed [3] (see below), but this must be complemented by HLA-C role, which is the only classical class I molecule expressed  at the cytotrophoblast and shows both presenting and suppressive functions [44].

## HLA-G

### Structure

Thirty-three different functional *HLA-G* alleles exist [41], and five ‘null’ alleles have been found (Fig. 2) [41, 61] of which only one, HLA-G*01:05N, has been found in more than one population and widespread around the World [38, 62, 63] (See “HLA-G*01:05N, -G*01:01 and -G*01:04 alleles World distribution: significance” section below). HLA-G proteins, like classical HLA class I molecules, are composed of a heavy chain, which is non-covalently bound to β2-microglobulin. *HLA-G* gene also shows similarity to the classical *HLA* loci, exhibits 7 introns and 8 exons, and encodes only for the heavy molecule, whereas β2-microglobulin is encoded for by a gene on chromosome 15 [4] (Fig. 2). Homo-dimeric HLA-G soluble isoforms have been described, like G2 and G6, and also heterodimeric isoforms associated with β2-microglobulin, like G1 and G5 [3, 64].

### Evolution

**Fig. 3:**
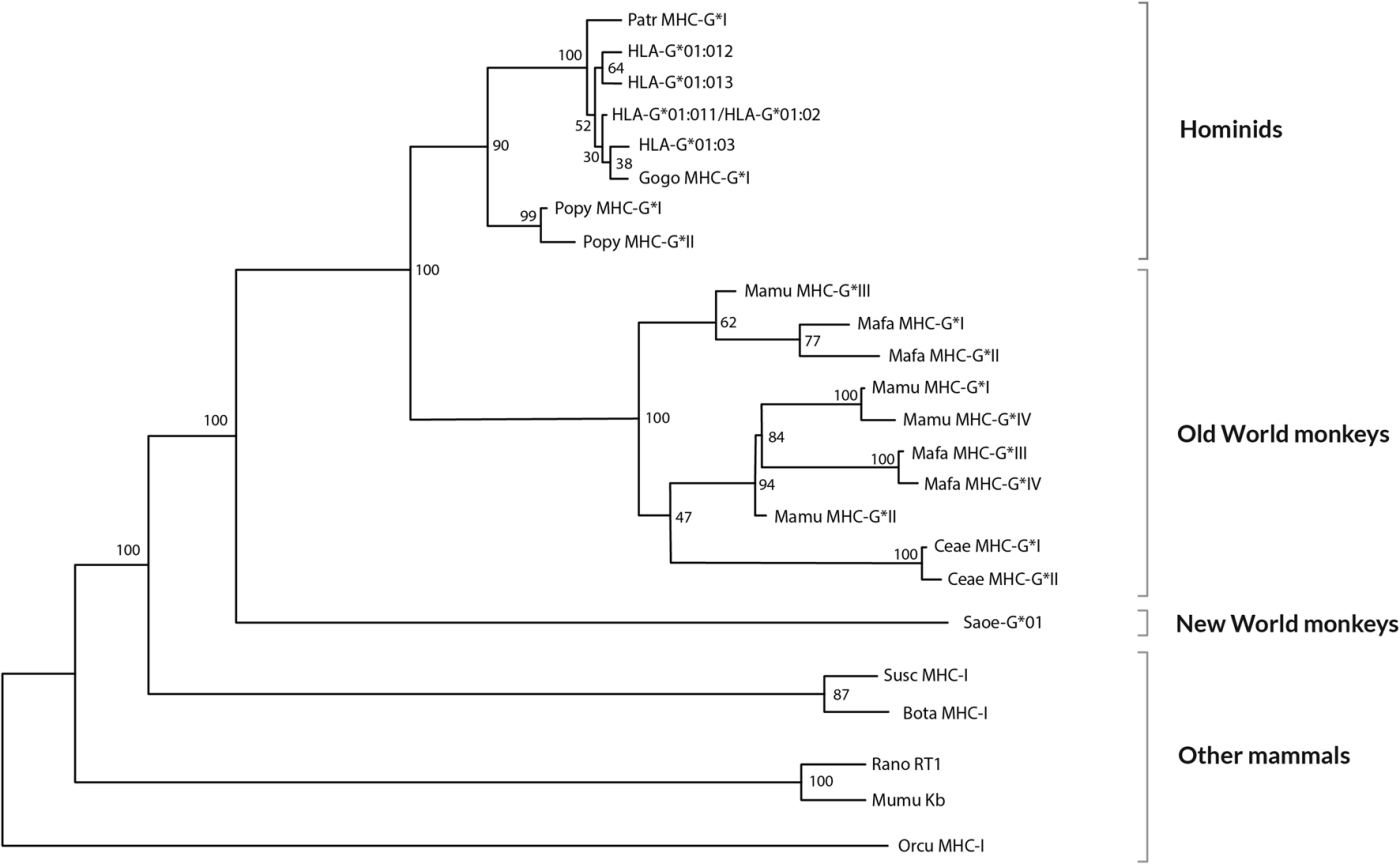
Relatedness Neighbor-Joining (NJ) dendrogram constructed with *MHC-G* exons1, 2, 3 and 4 sequences of man (HLA), chimpanzee (Patr), gorilla (Gogo), orangutan (Popy), rhesus monkey (Mamu), crab-eating macaque (Mafa), grivet (Ceae) and New World ape cotton-top tamarin (Saoe). It is shown that *MHC-G* of *Saguinus oedipus* diverges from all the other tested apes *MHC-G* [74]. Other mammals MHC-I sequences included in the analysis have been taken from GenBank: pig (Susc MHC-I; accession AF014002), cow (Bota MHC-I; accession X80936), mouse (MumuK^b^; accession U47328), rat (RanoRT1; accession X90376), and rabbit (Orcu MHC-I; accession K02441). Bootstrap values are shown

Parham et al. studies on classical *MHC* genes structure and evolution in apes should be consulted to better understand non-classical class I genes evolution [65, 66]. New World monkeys lineage separated about 35 million years ago [67, 68] from the lineage that gave rise to Old World and anthropoid monkeys. The cotton-top tamarin (*Saguinus oedipus*, *Saoe*) that inhabits Central-South America is a typical example of this group and has *MHC-G*-like genes instead of *MHC-A* and *MHC-B* genes [69]. However, *MHC-C* sequences have been also described in this New World monkey [70], which also binds KIR [71]. *MHC* of cotton-top tamarin shares more primary DNA sequence homologies with *HLA-G* than with classical class I* HLA* genes [69, 72, 73]. This is why, *MHC-G* has been assigned as the ancestral *MHC* class I gene and that *MHC* class I genes of the *Saoe* could be homologous to *HLA-G* genes. MHC-G is also present in Old World Monkeys, although MHC-E primary DNA structure may be closer to that of *Saoe* MHC [3] (see “HLA-E” section). The α1 domain of MHC-G molecule is preserved in all species studied (Fig. 3) and may be sufficient for MHC-G function in the subfamily of *Cercopithecinae* monkeys (*Macaca mulatta, Macaca fascicularis, Cercopithecus aethiops*) [3]. All the *MHC-G* alleles of this subfamily bear stop codons (like some human individuals; see below in “HLA-G*01:05N, -G*01:01 and -G*01:04 alleles World distribution: significance” section, *HLA-G* null alleles frequencies distribution) in a very restricted area of exon 3 (at codon 164), and some alleles may also show stop signals at codons 133, 118, and 176 [74]. However, pregnancies are normal in these *Cercopithecinae* species and functional MHC-G molecules may exist lacking the α2 domain, because one of the most important roles of MHC-G is preserving the fetus from maternal NK cells attack. Otherwise, reading-through stop codon mechanisms may exist [75]. *MHC-G* polymorphism is low in the *Pongidae* family: gorillas and chimpanzees [3, 76]. Intron 2 of *MHC-G* sequences show conserved motifs in all primate species: a 23-bp deletion starting in position 161, which is *MHC-G* locus specific. Surprisingly, the *Saoe MHC-G* intron 2 does not bear this deletion. Explanations for this finding could be that: (1) the *MHC-G*-like sequences in *Saoe* described did not give rise to the Old World monkey and human *MHC-G* alleles; or (2) the 23-bp deletion most likely occurred after separation of the New World monkeys from Old World monkey lineages about 35 million years ago [68, 69]. The first hypothesis is more plausible, since eluted peptides from cotton-top tamarin MHC-G like molecules are not typical of MHC-G [77]. *MHC-G* orthology has been studied by simple resemblance phylogenetic comparisons. However, lineal time inferences of species separation may be wrong and interpretation needs caution: this is because of  the frequent birth and death processes of genes and/or parts of them observed in the MHC region. Also, *MHC-G* in New World monkeys turns up as paralogous rather than orthologous to other primate *MHC-G* genes by cladistic studies on Alu and L1 elements insertions at 5’ region [78]. Indeed, this cladistic analysis concluded that *MHC-B* and *MHC-G* genes are ancestral to other *MHC* class I genes.

On the other hand, it has been found that MHC-G4, G5, and G6 isoforms are not present in gorilla, chimpanzee, and orangutan [76]. This finding suggests that MHC-G4 and the G5 and G6 soluble isoforms may be human-specific, and that *MHC-G* could have evolved independently in each group of primate species. With regard to these new findings, they make more difficult to assign a universal function for primate MHC-G proteins at the placental level or even at controlling autoimmunity [76]. Also, it has been found that *MHC-G* polymorphism shows more differences in *Cercopithecinae* family and in *Pongidae* species: (1) *Cercopithecinae* family bears a stop codon at exon 3, which is absent in *Pongidae* family. The latter bears a stop codon in exon 6, like humans [74]. This variation was generated 33 million years ago when both *Cercopithecinae* and *Pongidae* families diverged [79, 80]; (2) exon 7 is not found in *MHC-G* transcripts in human and *Pongidae* species, but it is preserved in rhesus monkeys (*Cercopithecinae* family) *MHC-G* mature mRNAs [81]; (3) *MHC-G2* “short” unusual splice variants have been found in *Gorilla* (*Pongidae*) and also in rhesus monkeys (*Cercopithecinae*) [76]. It seems that during the last 40 million years, a selective pressure has operated on MHC-G protein binding domain (antigen cleft, at exons 2 and 3) in New World and Old World primates and also in humans [15, 16].

In summary, it is striking that: (a) HLA-G*01:05N homozygous individuals there exist (non-functional HLA-G1 membrane-bound isoform) [82]; (b) MHC-G4, G5, and G6 isoforms are not necessary for survival in *Pongidae* family [76]; (c) *Cercopithecinae* family bears a stop codon at exon 3 [74]. These observations may lead to the conclusion that MHC-G is not a functional protein in Old World monkeys or may be substituted by other molecules [3, 64].

Moreover, presence of different HLA-G proteins in different primate species may be evolutionary better explained by mutations (i.e., deletions) that occurred at different apes speciation times. See reference [68], Fig. 3.

### DNA transcription and translation

*HLA-G* exon 1 encodes for the signal peptide. Exons 2, 3, and 4 transcribe for extracellular α1, α2, and α3 domains, respectively; and exons 5 and 6 for the transmembrane and the heavy chain cytoplasmic domain. HLA-G has a short cytoplasmic domain, because there exist a premature stop codon in exon 6; thus, exons 7 and 8 are not transcribed in the mature mRNA [4, 5].

### Surface molecules

Seven *HLA-G* transcripts produced by alternative mRNA splicing exist. Four of them give rise to membrane-bound protein isoforms and there are also three soluble isoforms [83]. HLA-G1 isoform is a complete HLA class molecule, with β2-microglobulin association. HLA-G2 lacks the α2 domain encoded for by exon 3 (Fig. 1), and HLA-G3 isoform has neither α2 nor α3 domains, encoded by for exons 3 and 4, respectively (Fig. 1). HLA-G4 does not have α3 domain, encoded by for exon 4. HLA-G5 and HLA-G6 soluble isoforms have the same domains than those of HLA-G1 and HLA-G2 isoforms; they are originated by transcripts which preserve intron 4, hindering the translation of the transmembrane domain (exon 5) (Fig. 1). Intron 4 is translated up to a stop codon in its 5’region; this is the cause that HLA-G5 and HLA-G6 isoforms to have a tail of 21 amino acids accounting for their solubility. HLA-G7 isoform has only the α1 domain together with two amino acids coded by intron 2, which is transcribed [83] (Fig. 1).

### Receptors

HLA-G extracellular domains bind to the following leukocyte receptors: CD8, LILRB1 and LILRB2 and the killer-cell immunoglobulin-like receptor KIR2DL4 (CD158d) (see Table 1). LILRB1 and LILRB2 also interact with the HLA-G molecule α3 domain and β2-microglobulin, LILRB2 having a higher affinity than LILRB1 for the molecule [3]. LILRB-binding sites are different for each receptor [3]. CD8 molecule also interacts with all MHC class I molecules through α3 domain of classical and non-classical MHC-I molecules, like HLA-G and HLA-E. CD8α/α binds to HLA-G with higher affinity, and with a lower affinity to HLA-E [3]. Moreover, β-2 microglobulin binds HLA-G isoform dimers (G1 and G5) and interacts with LILRB1 and LILRB2 receptors; LILRB1 predominantly binds β2-microglobulin-associated isoforms, while LILRB2 preferentially contacts β2-microglobulin-free HLA-G. Ability of HLA-G isoforms to associate in homodimers and their binding affinity depending on the receptor are important for HLA-G function [64, 84, 85].

**Table 1:** HLA-G, -E and -F receptors

Molecule	Receptor	References
HLA-G	LILRB1^a^	[64, 86, 88]
	LILRB2^b^	
	CD8^c^	
	KIR2DL4^d^	
HLA-E	CD94/NKG2A^e^	[87, 89]
	CD94/NKG2C^f^	[90]
	CD94/NKG2E^g^	
	TCR^h^	[48, 87]
	CD8^i^	[64]
	LILRB1^j^	[91]
	LILRB2^k^	
HLA-F	KIR3DL2^l^	[92, 93]
	KIR2DS4^m^	
	KIR3DS1^n^	[93]
	LILRB1^o^	[47, 91, 94]
	LILRB2^p^	[91, 94]

^a^Structure of this interaction has been defined by X-ray crystallography [95]

^b^Structure of this interaction has been defined by X-ray crystallography [14]

^c^Structure of this interaction has been defined by homology with crystallographic HLA-A2–CD8 and H-2 Kb–CD8 studies [86, 96, 97]

^d^Bibliography about structure of this interaction has not been found. Only functional assays using monoclonal antibodies have been used to discuss this interaction [11, 88, 98]

^e^Structure of this interaction has been defined by X-ray crystallography [99]

^f^Structure of this interaction has been defined by homology with crystallographic HLA-E–NKG2A studies [99]

^g^Structure of this interaction has been defined by homology with crystallographic HLA-E–NKG2A studies [99]

^h^Structure of this interaction has been defined by X-ray crystallography [100]

^i^Structure of this interaction has been defined by homology with crystallographic HLA-A2–CD8 and H-2 Kb–CD8 studies [86, 96, 97]

^j^Bibliography about structure of this interaction has not been found. Only affinity studies have been used to discuss this interaction [91]

^k^Bibliography about structure of this interaction has not been found. Only affinity studies have been used to discuss this interaction [91]

^l^Bibliography about structure of this interaction has not been found. Only functional assays using monoclonal antibodies have been used to discuss this interaction [92]

^m^Bibliography about structure of this interaction has not been found. Only functional assays using monoclonal antibodies have been used to discuss this interaction [92, 101]

^n^Bibliography about structure of this interaction has not been found. Only studies of interactions measured by surface plasmon resonance have been used to discuss this interaction [102]

^o^Structure of this interaction has been defined by X-ray crystallography [47]

^p^Bibliography about structure of this interaction has not been found. Only affinity studies have been used to discuss this interaction [102]

### Cellular interactions

HLA-G recognizes NK, T and B cells bearing the LILRB1 receptor on their surface [64]. Antigen presenting cells recognize both placental leucocytes and HLA-G + cells, which express LILRB1, and LILRB2 receptors. Also, HLA-G modulates NK cell cytotoxic activity in contact with LILRB1, LILRB2, and KIR2DL4 receptor [86–88]. Moreover, LILRB2 receptor in antigen presenting cells and CD8 receptor in CTL cells are recognized by HLA-G [87].

### HLA-G*01:05N, -G*01:01 and -G*01:04 alleles World distribution: significance

**Fig. 4:**
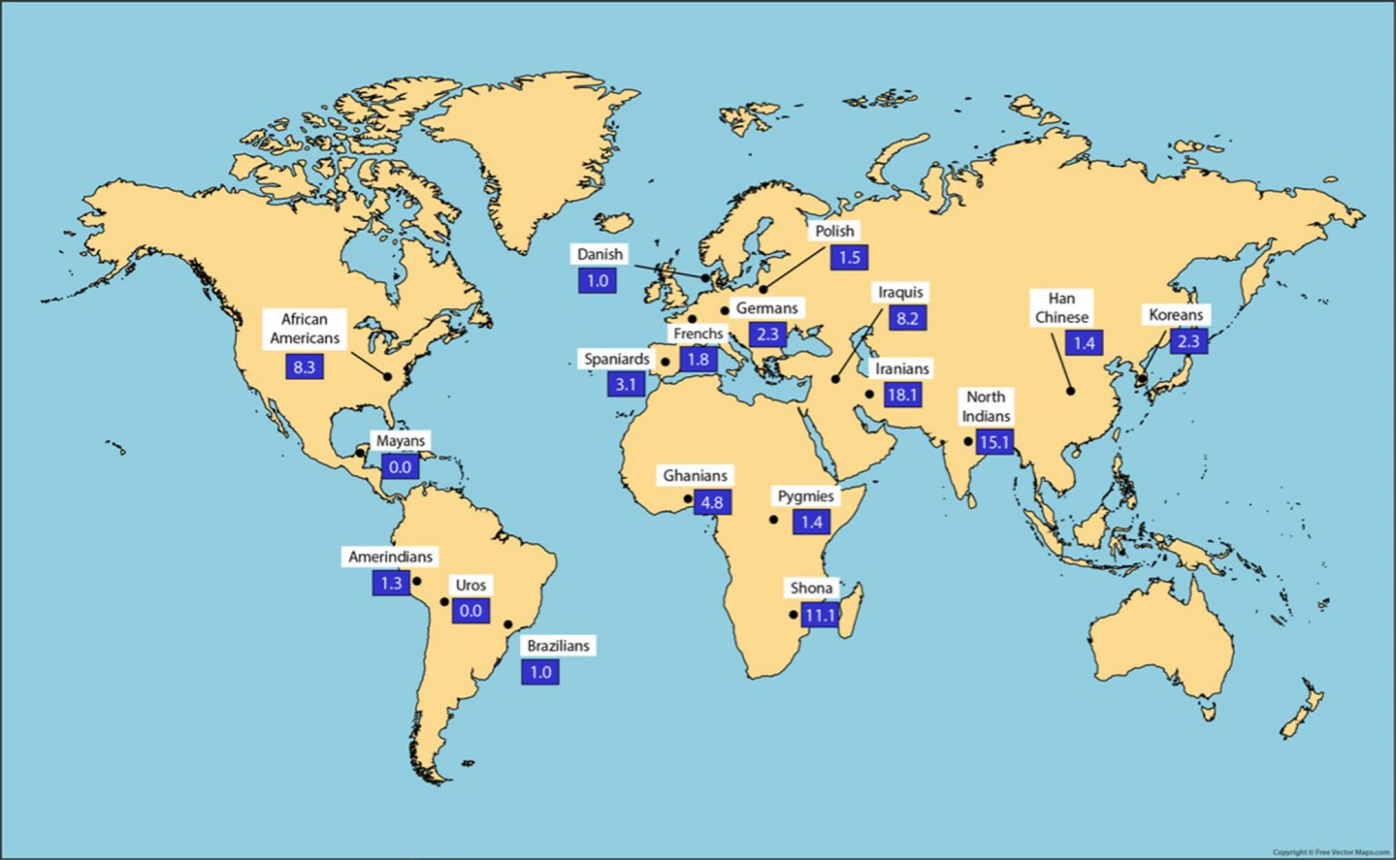
World map showing HLA-G*01:05N null allele frequencies in different populations. Populations are within white squares and HLA-G*01:05N frequencies are within blue squares. Note highest frequencies at Middle East (see text) [63]

The first confirmed *HLA-G* null allele was described by Arnaiz-Villena et al. in a Spanish population sample [38]. This *HLA-G* null allele protein could exist only with a single α1 domain: a single-base deletion induces a shift in the reading frame and a consequent premature stop codon. [3, 29, 39]. A protective effect against gestational infections has been associated with this allele but also recurrent spontaneous abortions [3, 64]. However, the hypothesis that frequent intrauterine infections can maintain high null allele frequencies is discarded, since Mayas and Uros populations, with a weaker health care services in comparison with European ones, do not have this allele. Also, Brazilian and mixed Amerindian populations show similar low frequencies [103]. Middle East Caucasians (Iraqis, Iranians, and Indians from North India) and some African populations (Ghana, Shona, and African Americans) show significantly higher frequencies of this null allele (Fig. 4). HLA-G*01:05N allele DNA sequence indicates that it was probably originated from the HLA-G*01:01 allele: both protein sequences are identical except for a cysteine deletion at codon 129/130 [82]. Moreover, HLA-G* 01:05N allele is in linkage disequilibrium with the HLA-A*30:01-B*13:02 haplotype, which is prevalent in Middle East and some Mediterranean populations. This haplotype may have been introduced in Spain by Muslim invaders in the eighth century AD or long before, when Saharan migrations took place from Saharan Desert to the Mediterranean Basin due to hyperarid climatic conditions beginning about 10,000–6,000 years ago [38, 104–107]. HLA-G*01:05N “founder effect” could place Middle East as the origin of this allele, because it contains the highest World reported frequencies [62].

As *HLA-G* is known to play an important role in maternal–fetal tolerance, it is striking how there exist HLA-G*01:05N healthy homozygous mothers capable of giving birth to normal and healthy fetuses. This finding indicates that the HLA-G1 isoform is not crucial for normal pregnancy development [82]. This is also supported by genus *Macaca* primates which have a normal development during pregnancy and adult life with HLA-G incomplete molecules [108, 109]. HLA-G α1 domain could be sufficient for the normal functioning of the HLA-G molecule, so negative evolutionary pressures would not act to eliminate this gene [39] or could be substituted by other HLA class I molecule at the placenta level. Also, HLA-G*01:05N allele may improve the level of immune response against HIV infection [110] or other infections not directly related to pregnancy.

**Fig. 5:**
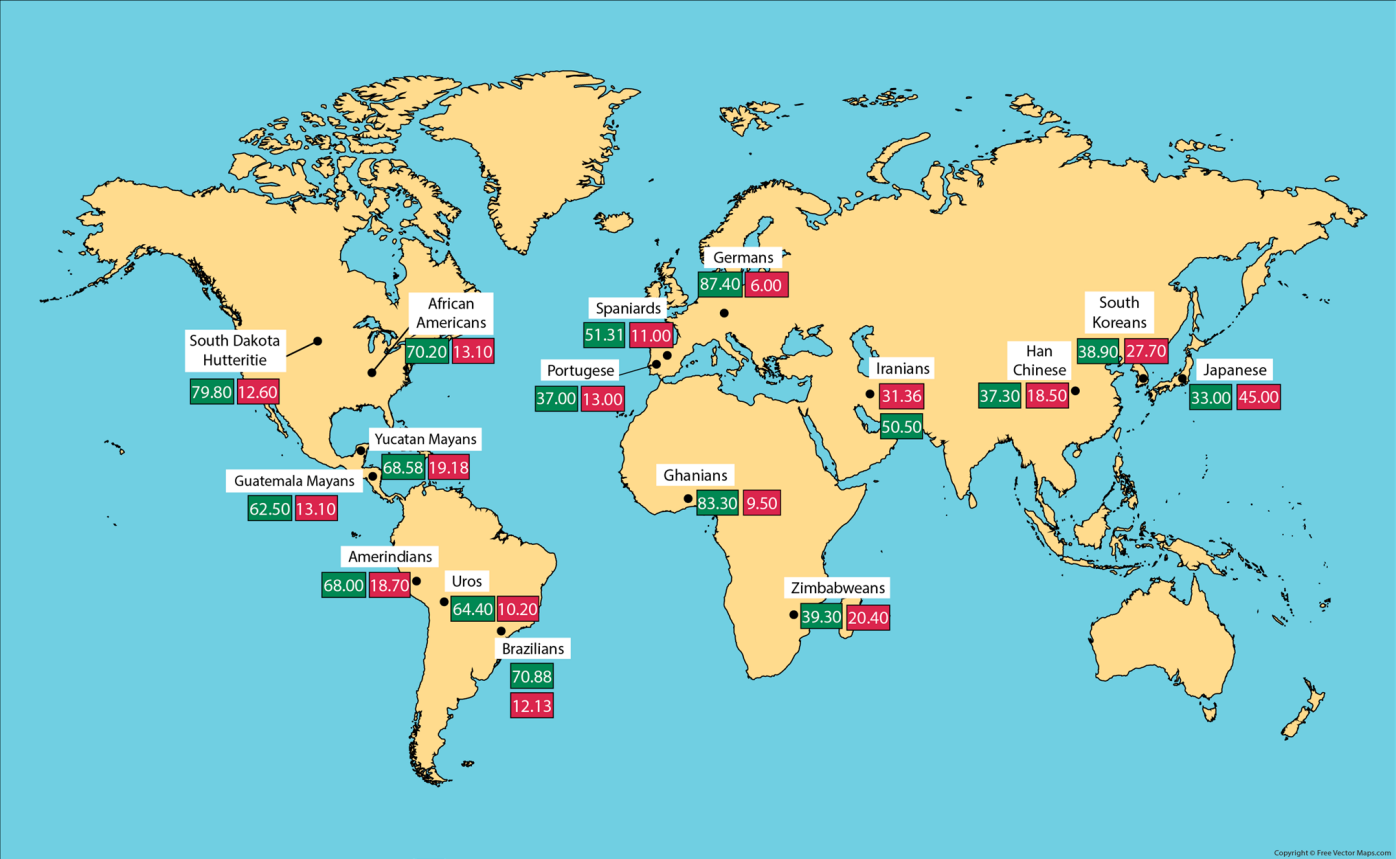
(1) HLA-G*01:04 frequencies (red squares) are different over the World. Higher frequencies are found in Japanese, Iranians, and South Koreans; Europeans and Amerindians show lower frequencies. (2) HLA-G*01:01 frequencies (green squares) do not clearly differ among World populations [63]

**Fig. 6:**
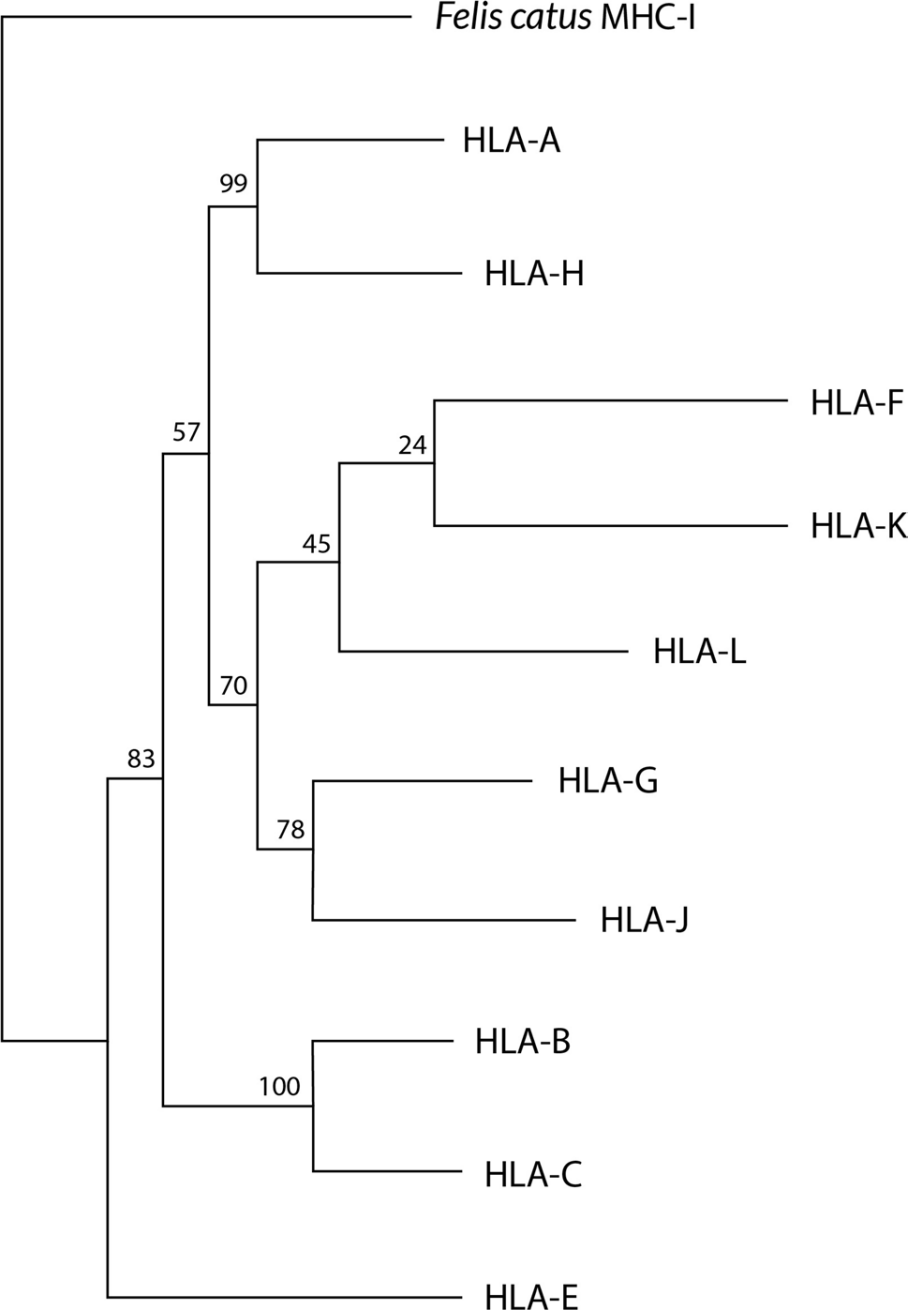
A Neighbor-Joining dendrogram showing that HLA-E may be the most ancient MHC molecule in humans. HLA sequences have been taken from IMGT/HLA database [41] and *Felis catus* MHC-I (GenBank accession NM_001305029.1) has been taken as outgroup. Bootstrap values are shown

On the other hand, highest frequencies of HLA-G*01:04 allele are found in South Korean, Iranian, and Japanese populations (27.7%, 31.36%, and 45%, respectively) (Fig. 5). Amerindian populations show similar HLA-G*01:04 allele frequencies among them: 10.2% in Uros from Titikaka Lake or 13.1% in Mayans from Guatemala. It is important to point out that HLA-G*01:04 allele frequencies higher than 10% have not been found in Europe neither higher than 13% in South Europe (Spaniards 11%, Portuguese 13%) (Fig. 5). Significant *HLA-G* differences have not been found, but a trend to lower frequencies in central Europe in comparison with Amerindians is detected (Fig. 6).

Also, higher frequencies of HLA-G*01:01 allele are found in USA South Dakota Hutteritie population, Ghanians, and Germans (79.8%, 83.3%, and 87.4%, respectively). Similar HLA-G*01:04 frequencies are found throughout all Amerindian populations (Fig. 5).

## HLA-E

### Structure

HLA-E is a heterodimer having an α heavy chain and a light chain (β-2 microglobulin). Heavy chain size is about 45 kDa and it is anchored to the cell membrane. *HLA-E* gene contains 8 exons. Exon 1 encodes for signal peptide, exons 2 and 3 encode for the α1 and α2 domains (peptide-binding site), exon 4 for the α3 domain, exon 5 for transmembrane domain, and exon 6 for cytoplasmic tail [111]. Exons 7 and 8 are not present in the mature mRNA.

### Evolution

Both New World and Old World monkeys MHC-E proteins preserve invariant residues at the tridimensional protein-presentation valve, like in all other MHC class I molecules from reptilians to humans. Also, the rate of substitutions in peptide-binding site reveals the exixtence of a high evolutionary pressure for stability in this area. *MHC-E* polymorphism in *Macaca mulatta* and *Macaca fascicularis* is restricted to 13 positions in exon 2 (3 synonymous and 10 nonsynonymous variations), 22 in exon 3 (10 synonymous and 12 nonsynonymous substitutions) and at the beginning of exon 4 (2 nonsynonymous variations); in contrast, exon 4 in humans does not show any variation in its sequence. Polymorphism in *MHC-E* gene of *Cercopithecus aethiops *is confined only to exon 3 with 1 synonymous and 1 nonsynonymous substitutions [112].

Regarding interspecific studies on *MHC-E*, an example of trans-specific *MHC-E* evolution has been found in genus *Macaca*: *Macaca mulatta* and *Macaca fascicularis* share the same *MHC-E* exon 2 and exon 3 sequence in one allele [112]: both Mamu-Mhc-E-*0101 and Mafa-Mhc-E*04 alleles are identical in exonic 2 and 3 sequences, only differing at the beginning of exon 4 at position 184 [112]. Also, a duplicated *MHC-E* locus has been found in *Macaca mulatta,* which may be originated by unequal crosses among different *MHC-E* homologue locus [113, 114]. These duplications have also been reported in other primates class II *MHC* genes but never before in class I loci [115]. On the other hand, these *Macaca mulatta* and *Macaca fascicularis* MHC-E protein alleles have a Tyrosine in position 36, where species of other different genera bear a Phenylalanine in this position (*Pongo pygmaeus, Cercopithecus aethiops, Homo sapiens*); this aminoacidic change in *Macaca* genus could have taken place in both species ancestor and confirms a trans-specific evolution of the *MHC* complex [112, 116, 117].

It was thought that *MHC-G* was primordial to other *MHC* genes in apes, giving rise to other typical *MHC* antigen presenting alleles, because it is present in New World monkeys (*Saguinus oedipus*), which are more ancient than Old World monkeys [16, 68, 118]. However, other data suggest that MHC-G molecules in primates could be non-functional (deletions in genus *Macaca*) [74] and other MHC proteins, like MHC-E, could do this function instead. It is known that MHC-G molecules in genus *Macaca* are not able to bind and present peptides and thus being surface expressed, because all individuals bear HLA-G deleted genes, but they may be useful for α1 interactions with cognate receptors [39, 74].

With regard to *Saguinus oedipus MHC-G* allele, it seems to be phylogenetically closer to *MHC-E* alleles of other species. These analyses were carried out using primary DNA sequences, genetic distances and Neighbor-Joining dendrograms that closely related *MHC-G* from New World primate (*Saguinus oedipus*) with MHC-E primary DNA sequences of macaque (*Macaca mulatta*) and orangutan (*Pongo pygmaeus*); it is also relevant that genus *Macaca* lack full *MHC-G* mRNA transcripts and DNA sequences [3, 74, 112, 118]. It has been shown that *HLA-E* locus is the most ancient HLA locus in humans, which may support the presence of MHC-E-like molecules in *Saguinus oedipus*, being the putative primitive MHC gene in primates [119] (Fig. 6).

It seems that selective pressures have occurred to conserve aminoacidic positions in the peptide-binding site of primate MHC-E molecules. It has been also found that *MHC-E* alleles have suffered trans-specific evolution, duplications, unequal crosses, and substitutions in primates, but it has remained for approximately 40 million years. Indeed, pockets of MHC-E presenting molecules among species, i.e., two human alleles, macaques MHC-E and MHC-E-like molecule in mouse (Qa-1b), have been studied and they all share main aminoacidic anchor portions during million years [120]. Also, human and medium-sized apes (macaques) MHC-E molecules present identical peptides to CD8 + T cells; in man, HLA-E presents leader peptides from class Ia molecules to regulate NK cells [121]. Other studies have also pointed out that *MHC-E* locus is the most conserved histocompatibility gene in primates, and this ancient evolutionary conservation of MHC-E peptide-binding site structure suggests a crucial relevance in immunological processes [112, 122].

### DNA transcription and translation

*HLA-E* transcripts are found in a great variety of tissues, and it is doubtful whether HLA-E molecules reach the surface in normal tissues conditions [123, 124]. However, scanty productive allelic changes described are mostly at the T-cell receptor-binding site [125], and it was put forward that HLA-E function may be related to the T-cell repertoire shaping in the thymus or otherwise to presenting a limited peptide repertoire. HLA-E is expressed in the cytoplasm and then on the surface of cytotrophoblast cells but only in the last months of the pregnancy and its expression control is mediated by INF gamma [126–128].

### Surface molecules

A nonamer peptide derived from residues 3–11 of signal sequences of most classical MHC class I molecules is required for HLA-E cell surface expression [28, 49, 129–132]. This leader peptide is released in the cytosol and then transported by TAP into the lumen of the endoplasmic reticulum, where it binds to HLA-E groove [49, 131]. Therefore, HLA-E surface expression allows NK cells to control the expression of a wide range of polymorphic MHC class I molecules through a single receptor. HLA-E surface expression inhibits NK-cell-mediated cytotoxicity [133].

### Receptors

ILT2 and ILT4 receptors bind to HLA-E [91]. It also has been shown to interact with other NK cell receptors like NKG2A [87, 132], NKG2C, and NKG2E [90]. Moreover, it is known that HLA-E can interact with TCR and CD8 receptors on the surface of CTL cells [48, 64, 87] (see Table 1).

### Cellular interactions

It was  detailed above that HLA-E regulates NK cell activity through interaction with  LILRB1, LILRB2, NKG2A, NKG2E, and NKG2C molecules : all of them are expressed on the NK cells surface [86, 87, 134, 135]. Also, HLA-E interacts with T CD8 + lymphocytes through TCR and CD8 [87].

## HLA-F

### Structure

HLA-F protein is a ~ 40–41 kDa molecule with HLA class I domains [136]. Due to an alternative splicing process, *HLA-F* mature mRNA does not contain the exon 7 sequence [137, 138], which leads to a modification in the protein, making cytoplasmic tail shorter in comparison to classical HLA class I proteins [137, 139] (see Fig. 1).

### Evolution

*HLA-F* orthologous DNA molecules are found in chimpanzee, bonobo, gorilla, and orangutan. Their amino acid sequences and their comparison with other primate MHC-F proteins show that MHC-F is a protein with a class I structure and that the characteristic residues of the peptide-binding region (PBR) are highly conserved in primate MHC-F. Therefore, MHC-F conservation along primate evolution suggests an important role in physiology. Thus, MHC-F protein could function together with MHC-G and MHC-E, in the natural killer (NK) cell activity regulation [140]. *HLA-F* orthologues have been compared in *Pongidae*, *Macaca* and American apes; they present only one pair of active *MHC-F* genes per chromosome whether they have or not duplicated genes. In addition, a New World (American) monkey, the marmoset, shows 6 orthologous transcripts. However, in all other New World monkeys, Old World ones and humans, *MHC-F* expansion by duplication has been inactivated to maintain only two parental *MHC-F* copies per individual irrespective of the number of duplicated copies contained: thus *MHC-F* gene is under purifying selection [141].

All *MHC-F* studies in chimpanzee, gorilla, orangutan, Rhesus macaque, and cotton-top tamarin have pointed out a mutation in intron 6 splice site, which drives to the lack of exon 7 in the mature MHC-F protein [69, 137–144]. This common characteristic among these species indicates that this  mutation took place before Old World and New World monkeys diverged about 35 million years ago [67, 68, 140].

*MHC-F* alleles in human, chimpanzee, bonobo, gorilla, and orangutan lack a six-nucleotide sequence that is present in rhesus macaque and cotton-top tamarin within exon 2. Thus, this six-nucleotide deletion happened in a common ancestor of genera *Homo*, *Pan*, *Gorilla* and *Pongo* after the separation of rhesus monkeys and New World monkeys’ evolutionary pathways. Phylogenetic trees performed show a strong similarity of *MHC-F* exons 2 and 4 sequences among species: all of them cluster together in a separated tree branch from other class I molecules [140].

Three-dimensional structure of HLA-F is similar to that of the other class I molecules. Also, the little differences observed among sequences of primate species indicate that there must be a strong selective pressure for invariance, except for the *Saguinus oedipus* (Saoe-F protein), that has a degree of difference of about 15%, while in the comparisons among other primates is under 6% [140].

### HLA-F transcription and translation

Molecules of HLA-F are intracellularly expressed in many body cells and tissues; these are peripheral blood lymphocytes (PBL), resting lymphocyte cells (B, T, NK), tonsils, spleen, thymus, kidney, brain, bladder, colon, liver, lymphoblast T-cell leukemia, and tumors. In addition, *HLA-F* is expressed on fetal extravillous trophoblast cells, which are in close contact with the maternal tissues [113]. *HLA-F* is expressed both intracellularly and on the surface of cytotrophoblast from the second trimester onwards [91, 118, 126, 145].

### HLA-F surface expression

Expression of HLA-F is found on the surface of activated lymphocytes, tumors, HeLa cells, EBV-transformed lymphoblastoid cells, and in some activated monocyte cell lines [89, 139]. HLA-F surface expression occurs after immune response activation: HLA-F is found on the surface of stimulated T memory cells but not on circulating regulatory T cells [146].

### HLA-F receptors

HLA-F tetramers have been shown to bind LILRB1 and LILRB2 receptors without any peptide binding [47, 64, 87–91, 94]. HLA-F open-conformed form has also been shown to bind KIR receptors of NK cells, like KIR3DL2 and KIR2DS4 [92, 93]. These HLA-F interactions are believed to stabilize other ligand–receptor interactions between trophoblast cells and decidual NK cells during pregnancy. Decidual NK cells play an important role in pregnancy immune regulation; binding to KIR2DS1 has also been shown [93] (see Table 1).

### Cellular interactions

It has been shown that HLA-F binds decidual NK cells in the trophoblast during pregnancy. It interacts with active or inactive NK cell activity in the maternal decidua through recognition of KIR3DL2, KIR2DS4 and KIR3DS1; these cells are also recognized through LILRB1 and LILRB2 receptors [87, 147]. Moreover, HLA-F recognizes T, B and NK cells which express LILRB1 receptor [64]. HLA-F + decidual leucocytes and antigen presenting cells interact also trough LILRB1 and LILRB2 receptors [64, 94].

## Conclusions

### Nature evolution vs statistical models

MHC was discovered in chicken by B. Briles in 1950 [148]. The first *HLA* and disease association was described by Amiel in 1967 [149]. Many diseases have been found statistically associated with *HLA* and *MHC* classical class I and class II genes. However, today, in 2022, no universally accepted pathogenesis mechanisms have been found to explain classical *HLA* genes and disease association [64] despite a flood of research on both statistical and  in vitro models trying to find out mechanisms and pathogenesis, suggesting pathogenetic proposals which are not yet universally accepted [64].

On the other hand, since Dan Geraghty [7] and Edgardo Carosella groups [150] uncovered HLA-G structure and immune system modulation by this molecule, another flood of *HLA-G* and disease studies has occurred, particularly in relation to autoimmunity, cancer, and fetal/maternal pathologies. Again, no mechanisms have been clarified up until now. In the meantime, *HLA-E* and *-F* immune suppressive genes have also been studied [128, 137]. It is then time to review and study on how Nature modulates the evolution of genes [151] at least in primates, where orthologous genes are well defined. This may give a clue on  function and associated pathology of these immune response control molecules, MHC-G, -E and -F. In this article, we have tried to shortly review some of these aspects.

### MHC genes for specific, non-specific, and regulatory immunity: extended HLA haplotypes

Much debate has occurred, because so many *MHC* different immune genes go close together in a short chromosome area across species from amniotes to humans during many million years [152–154]. This suggests that this set of genes may work together to save individual and species from external injuries, probably microbes, and associated self-recognition pathologies [3, 64, 155, 156]. In this chromosome region lies : a) non-adaptive immunity genes i.e.: C2, C4 and Bf complement factors, tumoral-necrotic factors (*TNF*) genes, heat shock proteins (*HSP*) genes, lymphotoxin genes (*LTA, LTB*) or some zinc finger codifying genes like *TRIM40*; b) adaptive immunity genes like tapasin (*TAP*) genes, lymphocyte antigen 6 (Ly6), *HLA* classical class I (*-A, -B, -C*), and class II (-*DQ, -DR, -DP*) genes or *MIC* genes (*MICA, MICB*); c) regulatory genes like HLA non-classical (*-G, -E, -F*) genes in primates, and others [157, 158]. Keeping together a set of certain alleles set of all known immune-related genes may be more advantageous for survival (i.e.: *MHC* haplotypes rather than single genes) [159] and this may be the reason why all these genes are transmitted conjointly at least from amniotes to humans [3, 64, 154, 158, 160]. A search as towhy they are transmitted and work together is worth to follow at this point of MHC/disease association nihilism. Coevolution of adaptive (i.e.: class I and class II), natural (i.e.: complement), and modulatory (i.e.: *HLA-G, HLA-E, HLA-F*) genes may point out that studying MHC haplotype/disease association in full or in part may be more fruitful to explain the association of HLA and disease than single-locus allele studies [161, 162].

### HLA haplotypes and disease association

Thus, the key for understanding HLA association to disease may be studying no single-locus genes but a cluster of neighboring and conjointly transmitted MHC genes (MHC haplotypes). It also would apply to HLA-G extended haplotypes and disease studies [64, 155, 156]. This approach was already suggested by Roger Dawkins in 1983 [161]: they tried to associate ankylosing spondylitis, rheumatoid arthritis, myasthenia gravis and systemic lupus erythematosus with complotypes (set of C2, Bf and C4 alleles inherited conjointly) and extended HLA haplotypes using different number of neighboring loci alleles. They also related susceptibility to diseases not only with HLA haplotypes but also with retroviruses inserted in the region, which affected expression of *MHC* genes and also their polymorphism and MHC segment duplication [162]. All or some of these factors within a complotype or a more extended haplotype should be studied to ascertain HLA and disease association. Indeed, this may be technically difficult to study but perhaps more fruitful. More or less long extended HLA haplotypes have been studied with some success in certain diseases; Berger’s Disease in 1984 [163], type I diabetes in 1992 [164], and some extended HLA haplotypes were also defined in 1991 [165]. However, relatively few studies have been done up until now; some of them were in microscopic polyangiitis [166], celiac disease [167], kidney disease [168, 169], diabetes [170], and psoriatic arthritis [171]. Technical difficulties of this type of study may be in part overcome by nowadays more advanced technologies.

### Additional remarks

1. MHC-G complete molecule is lacking in some humans and all primate individuals belonging to genus *Macaca*. Other MHC molecules may substitute its function or parts of the molecule may suffice for functionality.
2. Some apes do not have all of the MHC-G soluble isoforms described in man.
3. *MHC-E* (and not *-G*) may be the primordial *MHC* gene in apes, which gave rise to other MHC molecules.
4. A conjoint immune evolution and transmission in a relatively short DNA stretch of *MHC,* i.e.: immunosuppressive MHC genes (*MHC-G, -E, -F*), classical presenting molecules and non-adaptive ones (i.e.: C2, C4, Bf) is maintained for a long time from amniotes to human at least, because haplotypes or a specific set of MHC genes/alleles may be necessary for self-maintaining against pathogens and/or other injuries.

## Data Availability

Not applicable.

## References

[1] Klein J, Sato A (2000). The HLA system. First of two parts. N Engl J Med.

[2] Hviid TV (2006). HLA-G in human reproduction: aspects of genetics, function, and pregnancy complications. Hum Reprod Update.

[3] Donadi EA, Castelli EC, Arnaiz-Villena A, Roger M, Rey D, Moreau P (2011). Implications of the polymorphism of HLA-G on its function, regulation, evolution and disease association. Cell Mol Life Sci.

[4] Carosella ED, Moreau P, LeMaoult J, Rouas-Freiss N (2008). HLA-G: from biology to clinical benefits. Trends Immunol.

[5] Carosella ED, Favier B, Rouas-Freiss N, Moreau P, LeMaoult J (2008). Beyond the increasing complexity of the immunomodulatory HLA-G molecule. Blood.

[6] Berger DS, Hogge WA, Barmada MM, Ferrell RE (2010). Comprehensive analysis of HLA-G: implications for recurrent spontaneous abortion. Reprod Sci.

[7] Geraghty DE, Koller BH, Orr HT (1987). A human histocompatibility complex class I gene that encodes a protein with a shortened cytoplasmic segment. Proc Natl Acad Sci USA.

[8] Comiskey M, Goldstein CY, De Fazio SR, Mammolenti M, Newmark JA, Warner CM (2003). Evidence that HLA-G is the functional homolog of mouse Qa-2, the Ped gene product. Hum Immunol.

[9] Colonna M (1997). Specificity and function of immunoglobulin super- family NK cell inhibitory and stimulatory receptors. Immunol Rev.

[10] Ponte M, Cantoni C, Biassoni R, Tradori-Cappai A, Bentivoglio G, Vitale C (1999). Inhibitory receptors sensing HLA-G1 molecules in pregnancy: decidua-associated natural killer cells express LIR-1 and CD94/NKG2A and acquire p49, an HLA-G1-specific receptor. Proc Natl Acad Sci USA.

[11] Rajagopalan S, Long EO (1999). A human histocompatibility leukocyte antigen (HLA)-G-specific receptor expressed on all natural killer cells. J Exp Med.

[12] Gao GF, Willcox BE, Wyer JR, Boulter JM, O’Callaghan CA, Maenaka K (2000). Classical and nonclassical class I major histocompatibility complex molecules exhibit subtle conformational differences that affect binding to CD8 α/α. J Biol Chem.

[13] Contini P, Ghio M, Poggi A, Filaci G, Indiveri F, Ferrone S (2003). Soluble HLA-A, -B, -C and -G molecules induce apoptosis in T and NK CD8 (+) cells and inhibit cytotoxic T cell activity through CD8 ligation. Eur J Immunol.

[14] Shiroishi M, Kuroki K, Rasubala L, Tsumoto K, Kumagai I, Kurimoto E (2006). Structural basis for recognition of the nonclassical MHC molecule HLA-G by the leukocyte Ig-like receptor B2 (LILRB2/LIR2/ILT4/CD85d). Proc Natl Acad Sci.

[15] Moreno-Pelayo MA, Fernández-Soria VM, Paz-Artal E, Ferre-López S, Rosal M, Morales P (1999). Complete cDNA sequences of the DRB6 gene from humans and chimpanzees: a possible model of a stop codon readingthrough mechanism in primates. Immunogenetics.

[16] Morales P, Martinez-Laso J, Castro MJ, Gomez-Casado E, Alvarez M, Rojo-Amigo R, Kasahara M (2000). An evolutive overwiew of the MHC-G polymorphism: clues about the unknown function(s). The major histocompatibility complex: evolution, structure and function.

[17] Low SC, Berry MJ (1996). Knowing when not stop: selenocysteine incorporation in eukaryotes. Trends Biochem Sci.

[18] Stadtman TC (1996). Selenocysteine. Annu Rev Biochem.

[19] Amiot L, Vu N, Samson M (2015). Biology of the immunomodulatory molecule HLA-G in human liver diseases. J Hepatol.

[20] Scarabel L, Garziera M, Fortuna S, Asaro F, Toffoli G, Geremia S (2020). Soluble HLA-G expression levels and HLA-G/irinotecan association in metastatic colorectal cancer treated with irinotecan-based strategy. Sci Rep.

[21] Lee N, Malacko AR, Ishitani A, Chen MC, Bajorath J, Marquardt H (1995). The membrane-bound and soluble forms of HLA-G bind identical sets of endogenous peptides but differ with respect to TAP association. Immunity.

[22] Crisa L, McMaster MT, Ishii JK, Fisher SJ, Salomon DR (1997). Identification of a thymic epithelial cell subset sharing expression of the class Ib HLA-G molecule with fetal trophoblasts. J Exp Med.

[23] Mallet V, Blaschitz A, Crisa L, Schmitt C, Fournel S, King A (1999). HLA-G in the human thymus: a subpopulation of medullary epithelial but not CD83 (+) dendritic cells expresses HLA-G as a membrane-bound and soluble protein. Int Immunol.

[24] Le Discorde M, Moreau P, Sabatier P, Legeais JM, Carosella ED (2003). Expression of HLA-G in human cornea, an immune-privileged tissue. Hum Immunol.

[25] Menier C, Rabreau M, Challier JC, Le Discorde M, Carosella ED, Rouas-Freiss N (2004). Erythroblasts secrete the nonclassical HLA-G molecule from primitive to definitive hematopoiesis. Blood.

[26] Ito T, Ito N, Saathoff M, Stampachiacchiere B, Bettermann A, Bulfone-Paus S (2005). Immunology of the human nail apparatus: the nail matrix is a site of relative immune privilege. J Invest Dermatol.

[27] Cirulli V, Zalatan J, McMaster M, Prinsen R, Salomon DR, Ricordi C (2006). The class I HLA repertoire of pancreatic islets comprises the nonclassical class Ib antigen HLA-G. Diabetes.

[28] Braud V, Jones EY, McMichael A (1997). The human major histocompatibility complex class Ib molecule HLA-E binds signal sequence-derived peptides with primary anchor residues at positions 2 and 9. Eur J Immunol.

[29] Larsen MH, Hviid TV (2009). Human leukocyte antigen-G polymorphism in relation to expression, function, and disease. Hum Immunol.

[30] Solier C, Mallet V, Lenfant F, Bertrand A, Huchenq A, Le Bouteiller P (2001). HLA-G unique promoter region: functional implications. Immunogenetics.

[31] Moreau P, Flajollet S, Carosella ED (2009). Non-classical transcriptional regulation of HLA-G: an update. J Cell Mol Med.

[32] Hviid TV, Sorensen S, Morling N (1999). Polymorphism in the regulatory region located more than 1.1 kilobases 50 to the start site of transcription, the promoter region, and exon 1 of the HLA-G gene. Hum Immunol.

[33] Hviid TV, Rizzo R, Christiansen OB, Melchiorri L, Lindhard A, Baricordi OR (2004). HLA-G and IL-10 in serum in relation to HLA-G genotype and polymorphisms. Immunogenetics.

[34] Tan Z, Shon AM, Ober C (2005). Evidence of balancing selection at the HLA-G promoter region. Hum Mol Genet.

[35] Castelli EC, Mendes-Junior CT, Veiga-Castelli LC, Roger M, Moreau P, Donadi EA (2011). A comprehensive study of polymorphic sites along the HLA-G gene: implication for gene regulation and evolution. Mol Biol Evol.

[36] Martinez-Laso J, Herraiz MA, Penaloza J, Barbolla ML, Jurado ML, Macedo J (2013). Promoter sequences confirm the three different evolutionary lineages described for HLA-G. Hum Immunol.

[37] Castelli EC, Mendes-Junior CT, Deghaide NH, de Albuquerque RS, Muniz YC, Simoes RT (2010). The genetic structure of 3’ untranslated region of the HLA-G gene: polymorphisms and haplotypes. Genes Immun.

[38] Suárez MB, Morales P, Castro MJ, Fernández V, Varela P, Alvarez M (1997). A new HLA-G allele (HLA-G*0105N) and its distribution in the Spanish population. Immunogenetics.

[39] Le Discorde M, Le Danff C, Moreau P, Rouas-Freiss N, Carosella ED (2005). HLA-G*0105N null allele encodes functional HLA-G isoforms. Biol Reprod.

[40] Faucher MC, Louvanto K, Syrjänen S, Roger M (2018). Characterisation of the novel HLA-G null allele, HLA-G*01:21N, in Finnish individuals. HLA.

[41] Robinson J, Halliwell JA, Hayhurst JD, Flicek P, Parham P, Marsh SG (2015). The IPD and IMGT/HLA database: allele variant databases. Nucleic Acids Res.

[42] Clements CS, Kjer-Nielsen L, Kostenko L, Hoare HL, Dunstone MA, Moses E (2005). Crystal structure of HLA-G: a nonclassical MHC class I molecule expressed at the fetal-maternal interface. Proc Natl Acad Sci USA.

[43] Ferreira LMR, Meissner TB, Tilburgs T, Strominger JL (2017). HLA-G: at the interface of maternal-fetal tolerance. Trends Immunol.

[44] Papúchová H, Meissner TB, Li Q, Strominger JL, Tilburgs T (2019). The dual role of HLA-C in tolerance and immunity at the maternal-fetal interface. Front Immunol.

[45] Jiang H, Canfield SM, Gallagher MP, Jiang HH, Jiang Y, Zheng Z (2010). HLA-E–restricted regulatory CD8+ T cells are involved in development and control of human autoimmune type 1 diabetes. J Clin Invest.

[46] Romagnani C, Pietra G, Falco M, Millo E, Mazzarino P, Biassoni R (2002). Identification of HLA-E-specific alloreactive T lymphocytes: a cell subset that undergoes preferential expansion in mixed lymphocyte culture and displays a broad cytolytic activity against allogeneic cells. Proc Natl Acad Sci.

[47] Dulberger CL, McMurtrey CP, Hölzemer A, Neu KE, Liu V, Steinbach AM (2017). Human leukocyte antigen F presents peptides and regulates immunity through interactions with NK cell receptors. Immunity.

[48] Kraemer T, Blasczyk R, Bade-Doeding B (2014). HLA-E: a novel player for histocompatibility. J Immunol Res.

[49] Braud VM, Allan DS, O'Callaghan CA, Söderström K, D'Andrea A, Ogg GS (1998). HLA-E binds to natural killer cell receptors CD94/NKG2A. B and C Nature.

[50] Burrows CK, Kosova G, Herman C, Patterson K, Hartmann KE, Edwards DRV (2016). Expression quantitative trait locus mapping studies in mid-secretory phase endometrial cells identifies HLA-F and TAP2 as fecundability-associated genes. PLOS Genet.

[51] Song S, Miranda CJ, Braun L, Meyer K, Frakes AE, Ferraiuolo L (2016). Major histocompatibility complex class I molecules protect motor neurons from astrocyte-induced toxicity in amyotrophic lateral sclerosis. Nat Med.

[52] Garcia-Beltran WF, Hölzemer A, Martrus G, Chung AW, Pacheco Y, Simoneau CR (2016). Open conformers of HLA-F are high-affinity ligands of the activating NK-cell receptor KIR3DS1. Nat Immunol.

[53] Körner C, Altfeld M (2012). Role of KIR3DS1 in human diseases. Front Immunol.

[54] Goodridge JP, Burian A, Lee N, Geraghty DE (2010). HLA-F complex without peptide binds to MHC class I protein in the open conformer form. J Immunol.

[55] LeMaoult J, Le Discorde M, Rouas-Freiss N, Moreau P, Menier C, McCluskey J (2003). Biology and functions of human leukocyte antigen-G in health and sickness. Tissue Antigens.

[56] Crispim JC, Duarte RA, Soares CP, Costa R, Silva JS, Mendes-Junior CT (2008). Human leukocyte antigen-G expression after kidney transplantation is associated with a reduced incidence of rejection. Transpl Immunol.

[57] Amiot L, Ferrone S, Grosse-Wilde H, Seliger B (2011). Biology of HLA-G in cancer: a candidate molecule for therapeutic intervention?. Cell Mol Life Sci.

[58] Silva TG, Crispim JC, Miranda FA, Hassumi MK, de Mello JM, Simoes RT (2011). Expression of the nonclassical HLA-G and HLA-E molecules in laryngeal lesions as biomarkers of tumor invasiveness. HistolHistopathol.

[59] Carosella ED (2011). The tolerogenic molecule HLA-G. Immunol Lett.

[60] Santos KE, Lima TH, Felício LP, Massaro JD, Palomino GM, Silva AC (2013). Insights on the HLA-G evolutionary history provided by a nearby Alu insertion. Mol Biol Evol.

[61] Lajoie J, Jeanneau A, Faucher MC, Moreau P, Roger M (2008). Characterization of five novel HLA-G alleles with coding DNA base changes. Tissue Antigens.

[62] Arnaiz-Villena A, Enriquez-de-Salamanca M, Areces C, Alonso-Rubio J, Abd-El-Fatah-Khalil S, Fernandez-Honrado M (2013). HLA-G(∗)01:05N null allele in Mayans (Guatemala) and Uros (Titikaka Lake, Peru): evolution and population genetics. Hum Immunol.

[63] Arnaiz-Villena A, Enriquez-de-Salamanca M, Palacio-Gruber J, Juarez I, Muñiz E, Nieto J (2018). HLA-G in amerindians: epidemiology and worldwide population comparison. Open Med J.

[64] Arnaiz-Villena A, Juarez I, Suarez-Trujillo F, López-Nares A, Vaquero C, Palacio-Gruber J (2020). HLA-G: function, polymorphisms and pathology. Int J Immunogenet.

[65] Parham P, Lawlor DA (1991). Evolution of class I major histocompatibility complex genes and molecules in humans and apes. Hum Immunol.

[66] Lienert K, Parham P (1996). Evolution of MHC class I genes in higher primates. Immunol Cell Biol.

[67] Pilbeam D (1984). The descent of hominoids and hominids. Sci Am.

[68] Castro MJ, Morales P, Martínez-Laso J, Allende L, Rojo-Amigo R, González-Hevilla M (2000). Evolution of MHC-G in humans and primates based on three new 3'UT polymorphisms. Hum Immunol.

[69] Watkins DI, Chen ZW, Hughes AL, Evans MG, Tedder TF, Letvin NL (1990). Evolution of the MHC class I genes of a New World primate from ancestral homologues of human non-classical genes. Nature.

[70] Alvarez-Tejado M, Martinez-Laso J, Garcia-de-la-Torre C, Varela P, Recio MJ, Allende L (1998). Description of two Mhc-C-related sequences in the New World monkey Saguinusoedipus. Eur J Immunogenet.

[71] Parga-Lozano C, Reguera R, Gomez-Prieto P, Arnaiz-Villena A (2009). Evolution of major histocompatibility complex G and C and natural killer receptors in primates. Hum Immunol.

[72] Corell A, Morales P, Martínez-Laso J, Martín-Villa J, Varela P, Paz-Artal E (1994). New species-specific alleles at the Primate MHC-G Locus. Hum Immunol.

[73] Watkins DI, Letvin NL, Hughes AL, Tedder TF (1990). Molecular cloning of cDNA that encode MHC class I molecules from a New World primate (Saguinusoedipus). Natural selection acts at positions that may affect peptide presentation to T cells. J Immunol.

[74] Castro MJ, Morales P, Fernández-Soria V, Suarez B, Recio MJ, Alvarez M (1996). Allelic diversity at the primate MHC-G locus exon 3 bears stop codons in all Cercophitecinae sequences. Immunogenetics.

[75] Fernandez-Soria VM, Morales P, Castro MJ, Suarez B, Recio MJ, Moreno MA (1998). Transcription and weak expression of HLA-DRB6: a gene with anomalies in exon 1 and other regions. Immunogenetics.

[76] Castro MJ, Morales P, Martínez-Laso J, Allende L, Rojo-Amigo R, González-Hevilla M (2000). Lack of HLA- G4 and soluble (G5, G6) isoforms in the higher primates, Pongidae. Hum Immunol.

[77] Diehl M, Munz C, Keilholz W, Stevanovic S, Holmes N, Loke YW (1996). Nonclassical HLA-G molecules are classical peptide presenters. Curr Biol.

[78] Sawai H, Kawamoto Y, Takahata N, Satta Y (2004). Evolutionary relationships of major histocompatibility complex class I genes in Simian Primates. Genetics.

[79] Sibley CG, Ahlquist JE (1984). The phylogeny of the hominoid primates as indicated by DNA-DNA hybridization. J Mol Evol.

[80] Martin RD (1993). Primate origins: plugging the gaps. Nature.

[81] Boyson JE, Iwanaga KK, Golos TG, Watkins DI (1996). Identification of the rhesus monkey HLA-G ortholog. Mamu-G is a pseudogene. J Immunol.

[82] Castro MJ, Morales P, Rojo-Amigo R, Martinez-Laso J, Varela P, García-Bertiano M (2000). Homozygous HLA-G* 0105N healthy individuals indicate that membrane-anchored HLA-G1 molecule is not necessary for survival. Tissue Antigens.

[83] Paul P, Adrian Cabestre F, Ibrahim EC, Lefebvre S, Khalil-Daher I, Vazeux G (2000). Identification of HLA-G7 as a new splice variant of the HLA-G mRNA and expression of soluble HLA-G5, -G6, and -G7 transcripts in human transfected cells. Hum Immunol.

[84] HoWangYin KY, Loustau M, Wu J, Alegre E, Daouya M, Caumartin J (2012). Multimeric structures of HLA-G isoforms function through differential binding to LILRB receptors. Cell Mol Life Sci.

[85] Shiroishi M, Kuroki K, Ose T, Rasubala L, Shiratori I, Arase H (2006). Efficient leukocyte Ig-like receptor signaling and crystal structure of disulfide-linked HLA-G dimer. J Biol Chem.

[86] Shiroishi M, Tsumoto K, Amano K, Shirakihara Y, Colonna M, Braud VM (2003). Human inhibitory receptors Ig-like transcript 2 (ILT2) and (ILT4) compete with CD8 for MHC class-I binding and bind preferentially to HLA-G. PNAS.

[87] Ishigami S, Arigami T, Okumura H, Uchikado Y, Kita Y, Kurahara H (2015). Human Leukocyte Antigen (HLA)-E and HLA-F expression in gastric cancer. Anticancer Res.

[88] Rajagopalan S, Long EO (2012). KIR2DL4 (CD158d): an activator receptor for HLA-G. Front Immunol.

[89] Lee N, Geraghty DE (2003). HLA-F surface expression on B cell and monocyte cell lines is partially independent from tapasin and completely independent from TAP. J Immunol.

[90] Kaiser BK, Barahmand-Pour F, Paulsene W, Medley S, Geraghty DE, Strong RK (2005). Interactions between NKG2x immunoreceptors and HLA-E ligands display overlapping affinities and thermodynamics. J Immunol.

[91] Allan DSJ, Lepin EJM, Braud VM, O’Callaghan CA, McMichael AJ (2002). Tetrameric complexes of HLA-E, HLA-F and HLA-G. J Immunol Methods.

[92] Goodridge JP, Burian A, Lee N, Geraghty DE (2013). HLA-F and MHC class I open conformers are ligands for NK cell Ig-like receptors. J Immunol.

[93] Sim MJW, Sun PD (2017). HLA-F: a new kid licensed for peptide presentation. Immunity.

[94] Apps R, Gardner L, Traherne J, Male V, Moffet A (2008). Natural-killer cell ligands at the maternal-fetal interface: UL-16 binding proteins, MHC class-I chain related molecules, HLA-F and CD48. Hum Reprod.

[95] Kuroki K, Matsubara H, Kanda R, Miyashita N, Shiroishi M, Fukunaga Y (2019). Structural and functional basis for LILRB immune checkpoint receptor recognition of HLA-G isoforms. J Immunol.

[96] Gao GF, Tormo J, Gerth UC, Wyer JR, McMichael AJ, Stuart DI (1997). Crystal structure of the complex between human CD8alpha(alpha) and HLA-A2. Nature.

[97] Kern PS, Teng MK, Smolyar A, Liu JH, Liu J, Hussey RE (1998). Structural basis of CD8 coreceptor function revealed by crystallographic analysis of a murine CD8alphaalpha ectodomain fragment in complex with H-2Kb. Immunity.

[98] Moradi S, Berry R, Pymm P, Hitchen C, Beckham SA, Wilce MC (2015). The structure of the atypical killer cell immunoglobulin-like receptor, KIR2DL4. J Biol Chem.

[99] Petrie EJ, Clements CS, Lin J, Sullivan LC, Johnson D, Huyton T (2008). CD94-NKG2A recognition of human leukocyte antigen (HLA)-E bound to an HLA class I leader sequence. J Exp Med.

[100] Sullivan LC, Walpole NG, Farenc C, Pietra G, Sum MJW, Clements CS (2017). A conserved energetic footprint underpins recognition of human leukocyte antigen-E by two distinct αβ T cell receptors. J Biol Chem.

[101] Lin A, Yan WH (2019). The emerging roles of human leukocyte antigen-F in immune modulation and viral infection. Front Immunol.

[102] Burian A, Wang KL, Finton KA, Lee N, Ishitani A, Strong RK, Geraghty DE (2016). HLA-F and MHC-I open conformers bind natural killer cell Ig-like receptor KIR3DS1. PLoS ONE.

[103] Castelli EC, Mendes-Junior CT, Donadi EA (2007). HLA-G alleles and HLA-G 14 bp polymorphisms in a Brazilian population. Tissue Antigens.

[104] Arnaiz-Villena A, de Palacio-Grüber J, Muñiz E, Campos C, Alonso-Rubio J, Gomez-Casado E (2016). HLA genes in Chimila Amerindians (Colombia), the Peopling of America and Medical implications. Int J Mod Anthrop.

[105] Arnaiz-Villena A, Muñiz E, del Palacio-Gruber J, Campos C, Alonso-Rubio J, Gomez-Casado E (2016). Ancestry of Amerindians and its impact in anthropology, transplantation, HLA pharmacogenomics and epidemiology by HLA study in Wiwa Colombian population. Open Med J.

[106] Arnaiz-Villena A, Ruiz-del-Valle V, López-Nares A, Suárez-Trujillo F (2021). Iberian inscriptions in Sahara Desert rocks (Ti-m Missaou, Ahaggar Mts. area, Algeria) and first evidence of incise Iberian rock scripts in continental North Africa. Int J Mod Anthrop.

[107] Arnaiz-Villena A, Ruiz-del-Valle V, López-Nares A, Suárez-Trujillo F (2021). The Northern Migrations from a drying Sahara (6,000 years BP): cultural and genetic influence in Greeks, Iberians and other Mediterraneans. Int J Mod Anthrop.

[108] Arnaiz-Villena A, Morales P, Gomez-Casado E, Castro MJ, Varela P, Rojo-Amigo R (1999). Evolution of MHC-G in primates: a different kind of molecule for each group of species. J Reprod Immunol.

[109] Aldrich C, Wambebe C, Odama L, Di Rienzo A, Ober C (2002). Linkage disequilibrium and age estimates of a deletion polymorphism (1597DeltaC) in HLA-G suggest non-neutral evolution. Hum Immunol.

[110] Lajoie J, Hargrove J, Zijenah LS, Humphrey JH, Ward BJ, Roger M (2006). Genetic variants in nonclassical major histocompatibility complex class I human leukocyte antigen (HLA)-E and HLA-G molecules are associated with susceptibility to heterosexual acquisition of HIV-1. J Infect Dis.

[111] NCBI (2021) https://www.ncbi.nlm.nih.gov/gene?Db=gene&Cmd=ShowDetailView&TermToSearch=3133. Accessed Sept 2021

[112] Alvarez M, Martinez-Laso J, Varela P, Diaz-Campos N, Gomez-Casado E, Vargas-Alarcon G (1997). High polymorphism of Mhc-E locus in non-human primates: alleles with identical exon 2 and 3 are found in two different species. Tissue Antigens.

[113] Corell A, Morales P, Varela P, Paz-Artal E, Martin-Villa JM, Martinez-Laso J (1992). Allelic diversity at the primate major histocompatibility complex DRB6 locus. Immunogenetics.

[114] Trtková K, Kupfermann H, Grahovac B, Mayer WE, O’hUigin C, Tichy H (1993). Mhc-DRB genes of platyrrhine primates. Immunogenetics.

[115] Slierendreg BL, Otting N, van Besouw N, Jonker M, Bontrop RE (1994). Expansion and contraction of rhesus macaque DRB regions by duplication and deletion. J Immunol.

[116] Klein J (1987). Origin of major histocompatibility complex polymorphism: the trans-species hypothesis. Hum Immunol.

[117] Klein J, Takahata N (1990). The major histocompatibility complex and the quest for origins. ImmunolRev.

[118] Gomez-Prieto P, Parga-Lozano C, Rey D, Moreno E, Arnaiz-Villena A, Mehra NK (2010). Chapter 9. HLA-G, -F and -E: polymorphism, function and evolution. The HLA complex in biology and medicine. A resource book.

[119] Parham P, Adams EJ, Arnett KL (1995). The origins of HLA-A, B, C polymorphism. Immunol Rev.

[120] Ruibal P, Franken KLMC, van Meijgaarden KE, van Loon J, van der Steen D, Heemskerk M (2020). Peptide binding to HLA-E molecules in humans, nonhuman primates, and mice reveals unique binding peptides but remarkably conserved anchor residues. J Immunol.

[121] Wu HL, Wiseman RW, Hughes CM, Webb GM, Abdulhaqq SA, Bimber BN (2018). The role of MHC-E in T cell immunity is conserved among Humans, Rhesus Macaques, and Cynomolgus Macaques. J Immunol.

[122] Knapp LA, Cadavid LF, Watkins DI (1998). The MHC-E locus is the most well conserved of all known primate class I histocompatibility genes. J Immunol.

[123] Ulbrecht M, Honka T, Person SE, Johnson JP, Weiss EH (1992). The HLA-E gene encodes two differentially regulated transcripts a cell surface protein. J Immunol.

[124] Ulbrecht M, Kellerman J, Johnson JP, Weiss EH (1992). Impaired intracellular transport and cell surface expression of non-polymorphic HLA-E: evidence of insufficient peptide binding. J Exp Med.

[125] Bjorkman PJ, Saper MA, Samraoui B, Bennet WS, Strominger JL, Wilery DC (1987). Structure of the human class I histocompatibility antigen, HLA-A2. Nature.

[126] Ishitani A, Sageshima N, Lee N, Dorofeeva N, Hatake K, Marquardt H (2003). Protein expression and peptide binding suggest unique and interacting functional roles for HLA-E, F, and G in maternal-placental immune recognition. J Immunol.

[127] Mizuno S, Trapani JA, Koller BH, Dupont B, Yang SY (1988). Isolation and nucleotide sequence of a cDNA clone encoding a novel HLA class I gene. J Immunol.

[128] Koller BH, Geraghty DE, Shimizu Y, DeMars R, Orr HT (1988). HLA-E. A novel HLA class I gene expressed in resting T lymphocytes. J Immunol.

[129] Braud VM, Allan DS, Wilson D, McMichael AJ (1998). TAP- and tapasin-dependent HLA-E surface expression correlates with the binding of an MHC class I leader peptide. Curr Biol.

[130] Borrego F, Ulbrecht M, Weiss EH, Coligan JE, Brooks AG (1998). Recognition of human histocompatibility leukocyte antigen (HLA)-E complexed with HLA class I signal sequence-derived peptides by CD94/NKG2 confers protection from natural killer cell-mediated lysis. J Exp Med.

[131] Lee N, Goodlett DR, Ishitani A, Marquardt H, Geraghty DE (1998). HLA-E surface expression depends on binding of TAP-dependent peptides derived from certain HLA class I signal sequences. J Immunol.

[132] Lee N, Llano M, Carretero M, Ishitani A, Navarro F, López-Botet M (1998). HLA-E is a major ligand for the natural killer inhibitory receptor CD94/NKG2A. Proc Natl Acad Sci.

[133] Tomasec P, Braud VM, Rickards C, Powell MB, McSharry BP, Gadola S (2000). Surface expression of HLA-E, an inhibitor of natural killer cells, enhanced by human cytomegalovirus gpUL40. Science.

[134] Borrego F, Masilamani M, Marusina AI, Tang X, Coligan JE (2006). The CD94/NKG2 family of receptors: from molecules and cells to clinical relevance. Immunol Res.

[135] Colonna M, Moretta A, Vély F, Vivier E (2000). A high-resolution view of NK-cell receptors: structure and function. Immunol Today.

[136] Wainwright SD, Biro PA, Holmes CH (2000). HLA-F is a predominantly empty, intracellular, TAP-associated MHC class Ib protein with a restricted expression pattern. J Immunol.

[137] Geraghty DE, Wei XH, Orr HT, Koller BH (1990). Human leukocyte antigen F (HLA-F). An expressed HLA gene composed of a class I coding sequence linked to a novel transcribed repetitive element. J Exp Med.

[138] O'Callaghan CA, Bell JI (1998). Structure and function of the human MHC class Ib molecules HLA-E, HLA-F and HLA-G. Immunol Rev.

[139] Boyle LH, Gillingham AK, Munro S, Trowsdale J (2006). Selective export of HLA-F by its cytoplasmic tail. J Immunol.

[140] Rojo R, Castro MJ, Martinez-Laso J, Serrano-Vela JI, Morales P, Moscoso J (2005). MHC-F DNA sequences in bonobo, gorilla and orangutan. Tissue Antigens.

[141] Otting N, de Groot NG, Bontrop RE (2020). Evolution of HLA-F and its orthologues in primate species: a complex tale of conservation, diversification and inactivation. Immunogenetics.

[142] Otting N, Bontrop RE (1993). Characterization of the rhesus macaque (Macaca Mulatta) equivalent of HLA-F. Immunogenetics.

[143] Lawlor DA, Warren E, Ward FE, Parham P (1990). Comparison of class I MHC alleles in humans and apes. Immunol Rev.

[144] Orr HT, Dupont B (1989). HLA class I gene family. Characterization of genes encoding non-HLA-A, -B, -C proteins. Immunobiology of HLA.

[145] Shobu T, Sageshima N, Tokui H, Omura M, Saito K, Nagatsuka Y (2006). The surface expression of HLA-F on decidual trophoblasts increases from mid to term gestation. J Reprod Immunol.

[146] Lee N, Ishitani A, Geraghty DE (2010). HLA-F is a surface marker on activated lymphocytes. Eur J Immunol.

[147] Gardiner CM (2007). Killer cell immunoglobulin-like receptors on NK cells: the how, where and why. Int J Immunogenet.

[148] Briles W, McGibbon W, Irwin DM (1950). On multiple alleles affecting cellular antigens in the chicken. Genetics.

[149] Amiel J, Teraski PI (1967). Study of the leukocyte phenotypes in Hodgkin’s disease. Histocompatibility testing.

[150] Carosella ED, Rouas-Freiss N, Paul P, Dausset J (1999). HLA-G: a tolerance molecule from the major histocompatibility complex. Immunol Today.

[151] Klein J (1986). Natural history of the major histocompatibility complex.

[152] Padian K, Chiappe LM, Currie PJ, Padian K (1997). Bird origins. Encyclopedia of Dinosaurs.

[153] Arnaiz-Villena A, Ruiz-del-Valle V, Reche P, Gomez-Prieto P, Lowy E, Zamora J (2010). Songbirds conserved sites and intron size of MHC class I molecules reveal a unique evolution in vertebrates. Open Ornithol J.

[154] Arnaiz-Villena A, Ruiz-del-Valle V, Muñiz E, Palacio-Gruber J, Campos C, Gómez-Casado E (2017). Major Histocompatibility Complex allele persistence in Eurasia and America in the genus Carduelis (Spinus) during million years. Open Ornithol J.

[155] Vaquero-Yuste C, Juarez I, Molina-Alejandre M, Molaes-López EM, López-Nares A, Suárez-Trujillo F (2021). HLA-G 3'UTR polymorphisms are linked to susceptibility and survival in Spanish gastric adenocarcinoma patients. Front Immunol.

[156] Martín-Villa JM, Vaquero-Yuste C, Molina-Alejandre M, Juarez I, Suárez-Trujillo F, López-Nares A (2022). HLA-G: too much or too little? Role in cancer and autoimmune disease. Front Immunol.

[157] Horton R, Wilming L, Rand V, Lovering RC, Bruford EA, Khodiyar VK (2004). Gene map of the extended human MHC. Nat Rev Genet.

[158] Miller M, Taylor RL (2016). Brief review of the chicken Major Histocompatibility Complex: the genes, their distribution on chromosome 16, and their contributions to disease resistance. Poult Sci.

[159] Degli-Esposti MA, Leaver AL, Christiansen FT, Witt CS, Abraham LJ, Dawkins RL (1992). Ancestral haplotypes: conserved population MHC haplotypes. Hum Immunol.

[160] Solinhac R, Leroux S, Galkina S, Chazara O, Feve K, Vignoles F (2010). Integrative mapping analysis of chicken microchromosome 16 organization. BMC Genomics.

[161] Dawkins RL, Christiansen FT, Kay PH, Garlepp M, McCluskey J, Hollingsworth PN (1983). Disease associations with complotypes, supratypes and haplotypes. Immunol Rev.

[162] Dawkins R, Leelayuwat C, Gaudieri S, Tay G, Hui J, Cattley S (1999). Genomics of the major histocompatibility complex: haplotypes, duplication, retroviruses and disease. Immunol Rev.

[163] Arnaiz-Villena A, Gonzalo A, Regueiro JR, Vicario JL, Ortuño J (1984). Extended HLA haplotypes and Berger’s disease. Clin Nephrol.

[164] Segurado OG, Iglesias-Casarrubios P, Morales P, Martinez-Laso J, Partanen J, Campbell RD (1992). Genetic structure of the novel low-frequency haplotype HLA-B49, SC01, DR4 and its contribution to insulin-dependent diabetes susceptibility. Immunogenetics.

[165] Segurado OG, Giles CM, Iglesias-Casarrubios P, Corell A, Martinez-Laso J, Vicario JL (1991). C4 Chido 3 and 6 distinguish two diabetogenic haplotypes: HLA-B49, SC01, DR4, DQw8 and B8, SC01, DR3, DQw2. Immunobiology.

[166] Tsuchiya N, Kobayashi S, Hashimoto H, Ozaki S, Tokunaga K (2006). Association of HLA-DRB1*0901-DQB1*0303 haplotype with microscopic polyangiitis in Japanese. Genes Immun.

[167] Sciurti M, Fornaroli F, Gaiani F, Bonaguri C, Leandro G, Di Mario F (2018). Genetic susceptibilty and celiac disease: what role do HLA haplotypes play?. Acta Biomed.

[168] Robson KJ, Ooi JD, Holdsworth SR, Rossjohn J, Kitching AR (2018). HLA and kidney disease: from associations to mechanisms. Nat Rev Nephrol.

[169] Pan Q, Ma X, Chen H, Fan S, Wang X, You Y (2019). A single center study of protective and susceptible HLA alleles and haplotypes with end-stage renal disease in China. Hum Immunol.

[170] Hajjej A, Almawi WY, Stayoussef M, Hattab L, Hmida S (2019). Association of HLA class II alleles and haplotypes type 1 diabetes in Tunisian Arabs. Exp Clin Endocrinol Diabetes.

[171] Cassia FF, Cardoso JF, Porto LC, Ramos-E-Silva M, Carneiro S (2021). Association of HLA alleles and HLA haplotypes with psoriasis, psoriatic arthritis and disease severity in a miscegenated population. Psoriasis (Auckl).

